# The Ethics of Taxing Sugar-Sweetened Beverages to Improve Public Health

**DOI:** 10.3389/fpubh.2020.00110

**Published:** 2020-04-16

**Authors:** Francisco Goiana-da-Silva, David Cruz-e-Silva, Oliver Bartlett, Joana Vasconcelos, Alexandre Morais Nunes, Hutan Ashrafian, Marisa Miraldo, Maria do Céu Machado, Fernando Araújo, Ara Darzi

**Affiliations:** ^1^Centre for Health Policy, Institute of Global Health Innovation, Imperial College London, London, United Kingdom; ^2^Faculdade de Ciências da Saúde, Universidade da Beira Interior, Covilhã, Portugal; ^3^Centre for Innovation, Technology and Policy Research, IN+, Instituto Superior Técnico, Universidade de Lisboa, Lisbon, Portugal; ^4^Department of Law, Maynooth University, Maynooth, Ireland; ^5^Centro Hospitalar Universitário São João, Serviço Nacional de Saúde, Oporto, Portugal; ^6^Centro de Administração e Políticas Públicas, Instituto Superior de Ciências Sociais e Políticas, Universidade de Lisboa, Lisbon, Portugal; ^7^Department of Economics and Public Policy & Centre for Economics and Policy Innovation, Imperial College Business School, London, United Kingdom; ^8^Faculdade de Medicina, Universidade de Lisboa, Lisbon, Portugal; ^9^Centro Hospitalar Universitário São João, Faculty of Medicine, Universidade do Porto, Oporto, Portugal; ^10^Department of Surgery and Cancer, Faculty of Medicine, Imperial College London, London, United Kingdom

**Keywords:** sweetened beverages, taxation, pricing policies, bioethics, health policy

## Abstract

The World Health Organization highlights fiscal policies as priority interventions for the promotion of healthy eating in its Action Plan for the Prevention and Control of Non-communicable Diseases. The taxation of sugar sweetened beverages (SSBs) in particular is noted to be an effective measure, and SSBs taxes have already been implemented in several countries worldwide. However, although the evidence base suggests that this will be effective in helping to combat rising obesity rates, opponents of SSBs taxation argue that it is illiberal and paternalistic, and therefore should be avoided. Bioethical analysis may play an essential role in clarifying whether policymakers should adopt SSBs taxes as part of wider obesity strategy. In this article we argue that no single ethical theory can account for the complexities inherent in obesity prevention strategy, especially the liberal theories relied upon by opponents of SSBs taxation. We contend that a pluralist approach to the ethics of SSBs taxation must be adopted as the only suitable way of accounting for the multiple overlapping, and sometimes, conflicting factors that are relevant to determining the moral acceptability of such an intervention.

## Key Messages

- Evidence shows that appropriately designed fiscal policies have the potential to impact diets and must be implemented through a concerted approach with other policy actions.
- The WHO identifies levying taxes on sugar-sweetened beverages as an effective measure to tackle NCDs.
- Bioethics may play an essential role in clarifying the acceptability of levying taxes on sugar-sweetened beverages.
- Arguments on the ethicality of levying taxes on sugar-sweetened beverages that are based solely on one ethical theory should be rejected.
- Use of a pluralist, principled account of public health ethics would better reflect the complexity of the policy decision at stake, and would produce more helpful conclusions on whether these novel public health interventions are ethical.

## Introduction

Bioethics is a multifaceted field of study. Even though it has evolved over recent decades, it is often mistaken as only representing medical ethics (1). Bioethics is not solely concerned with genetics, euthanasia and doctor-patient relationship, and must be transversal to all daily health related operations and sectors. Bioethics therefore has a role to play in the design and implementation of public health interventions to address key societal issues. One such issue is the design of interventions to prevent non-communicable diseases (NCDs), in particular fiscal policies (2).

Fiscal policies are outlined as a priority intervention in the promotion of healthy eating on the Action Plan for the Prevention and Control of NCDs in the WHO European Region 2016–2025, with taxes on sweetened beverages highlighted as an effective measure (3). Both taxes and subsidies are proven to influence purchasing behaviors, particularly in relation to sweetened beverages (4). Currently, taxation policies targeting the consumption of sugar-sweetened beverages (SSBs) are already implemented in several countries worldwide (Table 1).

**Table 1:** List of countries where taxation policies targeting the consumption of sweetened beverages have been implemented.

**Country**	**Introduced**	**Target**	**Type of tax**	**Rate of tax (US$)**
Norway	1924	Non-alcoholic beverages - Containing added sugar or sweeteners - Syrup concentrates	Specific excise	$0,40/L $2,43/L
	2019	Prepared products Concentrate (syrup) Concentrate (based on fruit, berries or vegetables without added sugar) Juice and Syrup based on fruit, berries or vegetables without added sugar		$0,56/L $3,42/L $1,19/L $0,20/L
Finland	1994 2011 2014	Sugar-sweetened beverages & juices Sugar-sweetened beverages & juices Sugar-free soft drinks, mineral waters Sugar-sweetened beverages & juices	Specific excise	$0,26/L $0,08/L $0,13/L $0,26/L
Ireland	1975–1992 2018	Soft drinks Non-alcoholic, water and juice based drinks with an added sugar content - Increased tax for drinks with sugar content > 8g/100ml - Normal tax for drinks with sugar content > 5g/100ml - Drinks with sugar content <5g/100ml	Specific excise	$0,73/L $0,34/L $0,23/L No tax
Chile	1979 2014	Alcoholic and non-alcoholic beverages Non-alcoholic beverages with added sugars and sweeteners - Sugary drinks containing >6.25 g sugar/100 ml - Sugary drinks containing <6.25 g sugar/100 ml - 100% fruit juice, dairy-based beverages and unflavoured water	Ad valorem excise	15% 13% 18% 10% No tax
Samoa	1984 2008	Soft drinks and carbonated beverages	Specific excise	$0,12/L $0,17/L
Northern Mariana Islands	1995	Sugar-sweetened beverages excluding milk, 100% fruit juices, water	Specific excise	$0,00014/L $0,005 per beverage container
American Samoa	2001	Soft drinks, non-alcoholic beverages or syrups	Specific excise	$0,42/L
French Polynesia	2002	Sweetened drinks	Specific excise	$0,38/L
Marshall Islands	2003	Imported carbonated drinks	Specific excise	$0,56/L
Fiji	2007 2011 2016	Non-alcoholic beverages with added sugars or sweeteners, powders and preparations, flavored and colored sugar syrups - Locally produced sweetened beverages - Imported powders and preparations - Locally produced sweetened beverages - Imported sweetened beverages	Specific excise Ad valorem excise Specific excise Ad valorem excise	$0,15/L 10% $0,17/L 10% 15 %
Nauru	2007	Imported products with added sugars, carbonated drinks	Ad valorem excise	30%
Hungary	2011	Sugar-added drinks, syrups or concentrates for soft drinks - Soft drinks with > 8g of sugar/100ml - Syrups or concentrates for soft drinks	Specific excise	$0,05/item $0,03/L $0,71/L
France	2012 2019	Non-alcoholic beverages with added sugar or artificial sweeteners Non-alcoholic beverages with - ≤ 1kg sugar/ 100ml - 10-11kg of added sugar /100mL - >15kg of added sugar /100ml	Specific excise	$0,85/L Sliding scale tax $0,034/L $0,18/L $0,27/L + $2,29/ kg of sugar added
Mauritius	2013 2016	Sugar sweetened non-alcoholic beverages Any non-alcoholic beverage containing sugar	Specific excise	$0,0008/g of sugar
Tonga	2013	Carbonated and other non-alcoholic beverages, with added sugars or sweeteners	Specific excise	$0,50/L
Cook Islands	2013 2014	Import duty on sweetened drinks Non-alcoholic beverages with added sugars	Ad valorem excise Specific excise	15% $6,78/kg of sugar
Kiribati	2014	Non-alcoholic beverages (drinks and fruit concentrates) with added sugar	Ad valorem excise	40%
Latvia	2014	Non-alcoholic beverages with added sugars or sweeteners	Specific excise	$0,086/L
Latvia	2014	Sugar-sweetened beverages (excluding milks or yogurts)	Specific excise	$0,053/L
USA St Helena (CA)	2014	Carbonated drinks with ≥15 g sugar/L	Specific excise	$1,00/L
Barbados	2015	Carbonated soft drinks, juices with added sugars and SSBs (exempts 100% juice, coconut water, and plain milk)	Ad valorem excise	10%
USA Berkeley (CA)	2015	Sugar-sweetened beverages (excluding meal-replacement and dairy drinks, diet sodas, fruit juice, and alcoholic beverages)	Specific excise	$0,34/L
The Navajo Nation	2015	Minimal-to-no nutritional value food items, including sugar-sweetened beverages (junk food tax)	Ad valorem excise	2%
Dominica	2015	Non-alcoholic beverages with added sugars or sweeteners (soft drinks and energy drinks) and foods with high sugar content	Ad valorem excise	10%
Vanuatu	2015	Carbonated beverages with added sugars or sweeteners	Specific excise	$0,47/L
Belgium	2016	All soft drinks with added sweeteners, any substance intended for the use of manufacturing soft drinks -Liquids - Powders	Specific excise	$0,08/L $0,47/L $0,78/100kg
Ecuador	2016	Non-alcoholic beverages and juices with <50% fruit content - Beverages with <25g of sugar/L - Beverages with >25g sugar/L	Ad valorem excise Specific excise	10% $0,18/100g of sugar
Brunei	2017	Sugar-sweetened beverages with >6g total sugar/100mL	Specific excise	$0,28/L
Bahrain	2017	Energy drinks Soft drinks	Ad valorem excise	100 % 50 %
Saudi Arabia	2017	Energy drinks Carbonated drinks	Ad valorem excise	100 % 50 %
Portugal	2017 2019	Drinks with sugar contents - <80 g/L of final product - 80 g/L of final product Drinks with sugar contents - <25g/L of final product - 25-50g/L of final product - 50-80g/L of final product - >80g/L of final product	Specific excise	$0,10/L $0,20/L $0,015/L $0,07/L $0,09/L $0,23/L
Spain (Cataluña)	2017	Non-alcoholic beverages with - 5–8g of sugar for every 100ml - > 8g of sugar for every 100ml	Specific excise	$0,09/L $0,14/L
United Arab Emirates	2017	Energy drinks Carbonated drinks (except sparkling water)	Ad valorem excise	100 % 50 %
USA Cook County (IL)	2017	Non-alcoholic beverages with added sugars or sweeteners	Specific excise	$0,34/L
USA Boulder (CO)	2017	Any non-alcoholic beverage which contains at least 5 grams of caloric sweetener per 12 fluid ounces	Specific excise	$0,68/L
USA Albany (NY)	2017	Non-alcoholic beverages with added caloric sweetener (exempts 100% juice, artificially sweetened or alcoholic beverages, infant formula, milk products, and medical drinks)	Specific excise	$0,34/L
USA Philadelphia (PA)	2017	Non-alcoholic beverages, syrups or concentrates sweetened by any form of caloric, sugar-based sweetener or any form of artificial-sugar substitute	Specific excise	$0,53/L
USA Oakland (CA)	2017	Sugar-sweetened beverages (exempts 100% juice, artificially sweetened or alcoholic beverages, infant formula, milk products and medical drinks)	Specific excise	$0,34/L
Thailand	2017	Drinks with added sugar and natural sugar, excluding non-sugar sweeteners -Beverages with > 6 g of sugar per 100 ml	Ad valorem excise (will increase in two phases)	20-30%
Sri Lanka	2017	Sweetened beverages	Specific excise (whichever is higher)	$0,003/g of sugar or $0,08/ L
India	2017	Beverage based on fruit pulp or juice Flavored and sweetened aerated waters	Ad valorem excise	12% 40%
USA Seattle (WA)	2018	Sugar- sweetened beverages (exempts diet sodas, milk-based products, & fruit juice)	Specific excise	$0,62/L
Bermuda	2018	Soft drinks, flavored waters, syrups containing sugar or other sweeteners and sugar confectionery not containing cocoa	Ad valorem excise	50%
USA San Francisco(CA)	2018	Sugar-sweetened beverages, syrups and powders, containing added sugar and > 25 calories per 12 ounces (excluding 100% juice, artificially sweetened or alcoholic beverages, infant formula, dairy products and medical drinks)	Specific excise	$0,34/L
South Africa	2018	Sugar- sweetened beverages with > 4g of sugar /100ml	Specific excise	$0,002/g of sugar
United Kingdom	2018	Non-alcoholic, water and juice based drinks with an added sugar content - Beverages with sugar content <5g/100ml - Normal tax for drinks with sugar content > 5g/100ml - Increased tax for drinks with sugar content > 8g/100ml	Specific excise	No tax $0,24/L $0,32/L
Philippines	2018	Sugar- sweetened beverages - Using sugar and artificial sweeteners - With high-fructose corn syrup	Specific excise	$0,12/L $0,24/L
Estonia	2018 2019	Non-alcoholic beverages - with sugar content of 5–8 g/ 100 ml or with only artificial sweeteners - with artificial sweeteners and 5–8 g sugar/100 ml - with sugar content of > 10 g /100 ml - with sugar content of > 9 g /100 ml	Specific excise	$0,12/L $0,24/L $0,36/L $0,36/L
Denmark	1930 2011 2014	Soft drink tax - Beverages with >0.5g of sugar per 100ml - Beverages with <0.5g of sugar per 100ml Denmark repealed the tax on soft drinks	Specific excise	$0,25/L $0,12/L $0,45/L

*Source: developed by the authors based on information collected from national health authorities*.

## Methodology for Analyzing the Ethics of SSBs Taxation

Bioethical analysis can make an important contribution to the public health policymaking process by establishing a coherent moral framework for why governments should or should not intervene in public health in certain ways. This perspective article seeks to demonstrate how we can ensure that the ethical analysis we perform is as sound as possible.

Prior to analyzing the ethicality of particular public health interventions, analysts must establish why a particular health issue should be a matter of public concern and require government intervention. Several approaches to address this query are possible—some have answered it using social contract theory (5), some with political science (6). Ruger has developed a particularly powerful argument for government intervention in health that combines virtue ethics and capability theory (7). Whichever approach is taken, high prevalence of obesity in a population is generally accepted as an issue that governments should take responsibility for addressing.

In such context, analysts must evaluate whether a particular form of government intervention should be used to address a public health issue. In this paper we focus on the question of whether taxes on SSBs should be used to contribute to obesity prevention policy. The analysis we employ is doctrinal in nature. This involves looking at ethical arguments as syllogisms—a type of reasoning in which conclusions are drawn from premises—and testing whether the conclusions drawn in these arguments are logically sound. Premises are usually statements drawn from ethical doctrine—for example, interfering with autonomy is always wrong (first premise), and sugar taxes interfere with consumer autonomy (second premise). A conclusion must be based on factually accurate premises to be considered valid. The factual accuracy of premises will be assessed by examining whether they are supported by epidemiological evidence.

We first focus on the common claims that SSBs taxation is wrong because drinking SSBs does not harm others, and because taxation restricts consumer choice, which are grounded in deontological ethical theory (under which ethicality is judged according to rules or duties relating to the nature of actions). By applying the findings of behavioral science to the generalized premises on which this reasoning is based, we will show that they cannot be considered factually accurate, and therefore valid ethical conclusions cannot be drawn from them. However, we also find that changing the ethical theory does not change this outcome—claims grounded solely in other ethical theories, for example constructivism (under which ethicality is judged not according to the nature of actions but according to their outcomes), are equally unable to reflect the complexity of the evidence base surrounding SSBs consumption, and are therefore equally likely to produce unsound ethical conclusions.

We will use this brief analysis to then argue that the only way to arrive at valid conclusions on the ethicality of SSBs taxation is to ground the premises of arguments in a plurality of ethical theories. The policy decision to tax SSBs is based upon a complex evidence base, and an assessment of the ethicality of this decision must take account of this evidence base. Since basing an ethical argument on only one ethical theory tends to mean it is based on factually inaccurate premises, we argue that grounding ethical analysis in several ethical theories will result in the premises of that analysis better reflecting the complexity of the evidence base surrounding SSBs taxation (and obesity prevention generally), thus leading to sounder ethical conclusions.

To locate the information needed to conduct the above analysis, we primarily used Google Scholar keyword searches (e.g., “ethics” + “theories” + “liberalism” + “autonomy” + “sugar sweetened beverages” + “taxation”) to identify academic papers related to the ethics of SSBs taxation, the ethics of public health taxes, and the ethics of public health generally. We also used Google Scholar keyword searches (e.g., “sugar sweetened beverages” + “taxation” + “policy” + “evidence” + “impact” + “health”) to identify epidemiological literature on the population health impact of SSBs taxation and health inequality tends related to SSBs consumption, as well as behavioral science studies relating to consumer behavior (e.g., “consumer” + “behavior” + “evidence” + “soda”). We also relied specifically on similar keyword searches of leading public health ethics journals such as: *Public Health Ethics*; *Ethics, Medicine and Public Health*; and *The Journal of Law, Medicine and Ethics*. To ensure that our information remained current, we limited our searches to academic and policy sources dating from 2000 to the present (excepting sources that spoke to historical trends or viewpoints).

## Libertarian Ethics and SSBs Taxation

Opponents of SSBs taxes make the general argument that they are illiberal and paternalistic, and thus wrong (8). Libertarians, in the context of public health, believe that government intervention can only be ethical when it enables individual freedom and sustains the conditions for autonomous agency, (9) and this generally means limiting government intervention to non-coercive measures, or at least those coercive measures that are absolutely necessary to prevent an individual being harmed by another. This view is a form of deontological ethical theory, which broadly holds that actions should be judged by whether the nature of the action itself can be considered morally worthy. Individuals establish the rules governing whether an action is morally worthy though critical and rational self-reflection. Thus, the autonomy of human thought, and the freedom to reason critically are essential to moral conduct—anything that limits this autonomy and freedom should itself be considered unethical.

Evidently, fostering every individual's agency to make healthy decisions is important to population sustainability, however as is clear from the seminal work by Geoffrey Rose (10) and continued by others (11), promoting good health within a society requires greater attention to be devoted to how factors affecting health impact upon groups of individuals, rather than individuals in isolation. Public health interventions must therefore be designed primarily with the collective as the beneficiary of the intervention, not the individual. This means that judging the ethicality of a public health intervention, such as a tax on SSBs consumption, purely on the basis of the extent to which it interferes with individual autonomy and freedom means that we are only judging part of the motivation to adopt that intervention, and even then not the most important motivation (12). In the paragraphs below, we show how this impacts the validity of the ethical conclusions drawn from arguments based on autonomy.

Opponents of SSBs taxation usually deploy JS Mills' harm principle, stating that public health interventions should only be made to prevent harm to others, and since SSBs consumption only directly harms the individual, taxing SSBs is unethical. However, the evidence base demonstrates that population SSBs consumption clearly does have negative effects on others—higher rates of population SSBs consumption are linked to higher rates of obesity which place greater pressure on health care resources (13). This affects resource allocation decisions, which necessarily have negative implications for some areas of health and social care and impact the experiences of other individuals. Moreover, the harm principle as originally articulated by Mill does not insist that externalities must be proximate to individuals to any specific degree—in fact Mill himself insisted that government intervention in certain areas, such as consumer product regulation, should not be governed by his ideas on liberty at all, but by his doctrine on free trade in which he sets out how governments should balance economic efficiency with public welfare protection (14).

Thus, both premises in the libertarian argument are factually inaccurate—liberal ethical doctrine does not insist that public health interventions can only prevent direct harm to others, and SSBs consumption does generate significant social burdens in addition to the harms suffered by individual consumers. The reasoning of this argument does not sufficiently account for the complex relationship between high population levels of SSBs consumption, rates of obesity, and healthcare resource allocation, and government responsibility for social protection. Thus, evaluated on the strength of its reasoning, the libertarian argument that SSBs taxation is unethical should be seen as unsound.

Opponents also usually deploy ideas of autonomy and free choice, arguing that consumer autonomy holds a privileged status in moral considerations, and should be given the highest priority in the formation of public health interventions. Since taxing SSBs substantially interferes with consumers' abilities to make autonomous and free choices, taxing SSBs is unethical. However, the evidence base tells us that factors such as the availability of healthy compared to unhealthy foods and beverages (15) and pricing strategies for SSBs (16) heavily shape the choices available to individuals before they even arrive in a consumption choice situation. It is clear that when an individual makes a consumption choice, that choice is not a pure translation of individual preferences, but the outcome of a complex mix of individual preferences, marketing messages, environmental conditions and psychological pressures. Some of these influences on consumers' choices occur simply because the consumer is a certain gender, or because they were born in a particular neighborhood, meaning that different consumers will make choices about consuming healthy or unhealthy products from vastly different starting points. This is unfair, and according to the weight of evidence on the effect that health inequalities have on burdens of disease (17), combatting health inequality should be an important consideration for governments when choosing public health interventions—perhaps more important even than protecting consumer autonomy in every situation.

Moreover, this argument presumes that making SSBs more expensive will have an impact upon consumer autonomy. As recent analysis on the issue points out, autonomy and freedom should be distinguished, and reducing an individual's freedom to buy SSBs at low prices does not necessary reduce their ability to make autonomous decisions on whether to buy SSBs at higher prices (18). Furthermore, it is not clear from the epidemiological evidence that consumers' autonomy is affected by SSBs taxes. Evidence from the UK suggests that there is actually strong support for SSBs taxation, which would unlikely be the case if consumers felt that their ability to make health decisions for themselves was being compromised. Perhaps one could argue that consumers' autonomy could be compromised without them realizing, but then this would seem to support the argument for SSBs taxation—if the way in which an individual thinks about their food decisions can be easily manipulated by subtle changes to the decision-making context without the individual ever realizing this, as behavioral and marketing evidence shows that it can (19), this would support affording the protection of autonomy a less privileged position in public health decision making, and instead privileging how policies can prevent consumer decisions from being manipulated.

Thus, both premises in the deontological argument based on autonomy are factually inaccurate—governments have more (and arguably more important) duties than simply to protect consumer autonomy, and SSBs taxation probably does not appear to influence consumer autonomy to a very large extent (and testing this more forensically would constitute an acknowledgment that consumer autonomy is a frail concept). The reasoning of this argument does not sufficiently account for the complex influences that environmental conditions and social inequalities have on SSBs consumption, nor for the fact that an autonomous consumer may actually welcome public health interventions. Thus, evaluated on the strength of its reasoning, the argument from autonomy that SSBs taxation is unethical should also be seen as unsound.

### A Pluralist Approach to the Ethics of SSBs Taxation

The analysis above demonstrates that basing arguments on the ethics of public health practice on one ethical theory leads to unsound conclusions. This outcome is the same no matter what ethical theory is used in place of deontology, even if it seems more suited to guiding community-oriented action. For example, utilitarianism, the leading variant of constructivism, holds that an action is ethical if the consequences maximize utility (often understood as the greatest happiness experienced by the greatest number of people). These idea influences the development of obesity policy at the highest levels in the form of cost-benefit analysis of interventions (20). However, reliance on utilitarian analysis alone also cannot produce logically sound ethical conclusions where SSBs taxation is concerned, because the premises of most utilitarian argument do not account well for complexity either, and therefore are factually inaccurate. In particular, utilitarian analysis does not account well for hidden discrimination and inequality (21), which confounds utilitarian calculations of happiness.

For example, a utilitarian argument might be that the happiness produced by the health consequences of increasing SSBs prices (decreased population consumption of SSBs, contributing to reduced rates of population obesity, particularly childhood obesity) would be greater than the unhappiness produced by the economic consequences of increasing SSBs prices (somewhat lower profits for the industry on SSBs, and the inconvenience of having to pay slightly more for SSBs). However, this does not account for the fact that lower socioeconomic groups consume proportionately more SSBs, spend a greater proportion of their income on them, and suffer greater rates of obesity (22). In such circumstances, it becomes very difficult to accurately calculate the happiness gained or lost by this population, not to mention the relative weight that should be attributed to this against the gains or losses of higher socioeconomic groups. Given that the regressive nature of SSBs taxation is another common objection raised by the industry, it is particularly important that ethical analysis be capable of properly accounting for the relationship between inequality, health and fiscal policy. Utilitarian constructivist arguments are therefore, like deontological arguments, unable on their own to produce sound ethical conclusions on SSBs taxation, since they also tend to be founded on factually inaccurate premises.

The ethical analysis of SSBs taxation therefore must be pluralist. Most new ethical theories tailored to a public health context adopt such a pluralist (or principled) approach, meaning that they identify a discrete set of different principles or values that are deemed important for ethical public health policy, and judge a public health intervention according to all of these principles simultaneously. Table 2 lists five of the most common principles found in new accounts of public health ethics, which combine a number of the traditional ethical ideas surveyed above.

**Table 2:** Public health ethics principles.

**Principle**	**Description**
Least infringement	Interventions should take the form that least restricts the liberty of individuals within the population
Effectiveness	Interventions should be based on evidence and be effective in achieving their objectives
Harm	Interventions should only restrict the liberty of individuals within the population to prevent behaviors causing harm to others
Transparency	Interventions should be made with the participation of the population and with all possible transparency
Progressive impact	Interventions should seek to reduce health and social inequalities

*Source: Adapted from Kass (23), Childress et al. (24), Lee (25)*.

The potential flaws of principled ethical theories must also be recognized—the most important is the potential lack of clarity in how principles should be prioritized if they conflict, and how strong an obligation they should impose (26). In the authors' opinion, fulfilling one principle does not necessarily exclude the fulfillment of another. In the case of rights-based ethical accounts, the enjoyment of one right can exclude the enjoyment of another, and competing rights must be balanced using additional principles such as proportionality. However, fulfilling a principle does not necessarily mean that other principles cannot be simultaneously fulfilled. As long as principles are selected to be coherent with each other, a process to balance principles is not a necessity. As long as all principles carry equal weight, the only necessity is a criterion to decide how many principles an intervention must fulfill to be ethically acceptable.

## Conclusions

Extensive evidence shows that implementing taxes on the consumption of SSBs generates significant health gains and has a positive impact on the reduction of premature mortality. The WHO has now produced detailed guidance on how to design fiscal policies relating to diet, responding to the manifest interest of many countries for more detailed advice on how to implement their international commitments to adopt effective measures to reduce premature mortality attributable to obesity (27). However, despite several countries having implemented such taxes, other countries continue to resist doing so (28). The rationale for this resistance is primarily economic in nature, however sometimes the issue of the perceived legitimacy of the measure is also raised.

Further bioethical debate in obesity policy is essential to ensure that policymakers are equipped with not just sound epidemiological evidence, but sound moral evidence on which they can base the implementation of polices that will shape healthier food environments. In this paper we sought to demonstrate that looking at the ethical legitimacy of interventions such as SSBs taxation through only one ethical lens is only likely to produce invalid ethical conclusions, since the premises on which many single-theory ethical arguments are based are ill-equipped to properly reflect the complexity of factors that must be considered in policy decisions implement SSBs taxes. A pluralist approach to the ethics of fiscal policies such as levying taxes on SSBs is therefore essential to produce a sound explanation of whether they are morally acceptable in a democratic society. Even though there are also certain issues inherent to pluralist ethical accounts of public health practice, their use is more likely to enable progress in ethical debates in the field of non-communicable disease prevention.

## Author Contributions

FG: Paper concept, design, drafting, critical analysis, and final approval. DC: Acquisition of data, Paper drafting, critical analysis, and final approval. OB: Paper concept, design, drafting, critical analysis and final approval. JV: Acquisition of data, Data analysis and final approval. AM: Acquisition of data, data analysis and final approval. HA: Paper concept, critical analysis and final approval. MM: Paper concept, critical analysis and final approval. MCM: Paper concept, critical analysis and final approval. FA: Paper concept, critical analysis and final approval. AD: Paper concept, critical analysis and final approval.

### Conflict of Interest

The authors declare that the research was conducted in the absence of any commercial or financial relationships that could be construed as a potential conflict of interest.
